# Variants in Exon 11 of MEF2A Gene and Coronary Artery Disease: Evidence from a Case-Control Study, Systematic Review, and Meta-Analysis

**DOI:** 10.1371/journal.pone.0031406

**Published:** 2012-02-21

**Authors:** Yan Liu, Wenquan Niu, Zhijun Wu, Xiuxiu Su, Qiujin Chen, Lin Lu, Wei Jin

**Affiliations:** 1 Department of Cardiology, Ruijin Hospital, Shanghai Jiao Tong University School of Medicine, Shanghai, China; 2 State Key Laboratory of Medical Genomics, Ruijin Hospital, Shanghai Jiao Tong University School of Medicine, Shanghai, China; College of Pharmacy, University of Florida, United States of America

## Abstract

**Background:**

Coronary artery disease (CAD) is the most common heart disease worldwide. Association of CAD with variants in the myocyte enhancer factor 2A (*MEF2A*) gene, the first identified CAD-causing gene, has attracted special attention but the results are controversial. We aimed to evaluate this genetic association via a case-control study and meta-analysis.

**Methodology/Principal Findings:**

We performed a case-control association study to investigate the relationship between variations in exon 11 of *MEF2A* gene and CAD in 1045 sporadic patients and 1008 controls enrolled angiographically among southern Chinese population, and then the data from this study were compared and discussed in a systematic review and meta-analysis with all available published studies on *MEF2A* gene and CAD. In total, eight variants were identified (21-bp deletion, CAG repeats, CCG repeats, a CCA deletion and four SNPs). No significant link was observed between the common (CAG)_n_ polymorphism and CAD, whereas the rare 21-bp deletion was detected only in five affected individuals. The meta-analysis of (CAG)_n_ polymorphism and CAD risk, including nine studies with 3801 CAD patients and 4020 controls, also provided no convincing evidence for the genetic association, even upon stratification by race (mainly Whites and Chinese). However, the 21-bp deletion was regarded as a potentially logical, albeit undetermined, candidate for CAD in the following systematic review.

**Conclusions/Significance:**

Our findings failed to demonstrate a correlation between (CAG)_n_ polymorphism with CAD, however, we concluded that the rare 21-bp deletion might have a more compelling effect on CAD than the common (CAG)_n_ polymorphism, and *MEF2A* genetic variant might be a rare but specific cause of CAD/MI.

## Introduction

Coronary artery disease (CAD) is a common complex disorder resulting from both genetic and environmental influences [1], [2], and it has become a major cause of death and disability in China. The role of genetic alterations and their impact on CAD susceptibility remains unclear and has attracted more attention. In the past three decades, genetic association studies and genome-wide linkage scans have revealed a considerable number of candidate loci and genes for CAD and myocardial infarction (MI) [3]–[7], but results are not often reproducible [8]–[10].

In 2003, a 7-amino acid deletion, caused by a 21-base pair (bp) coding sequence deletion in exon 11 of the myocyte enhancer factor 2A (*MEF2A*) gene, was reported as a causative mutation in a single large CAD/MI family of Scandinavia ancestry [11]. *In vitro* functional analysis indicated that the 21-bp deletion disrupted the nuclear localization of mature protein and decreased *MEF2A*-induced transcriptional activation. Thus this genetic imperfection might lead to a defective or abnormal vascular endothelium, which could promote the genesis of atherosclerotic plaque or thrombosis and influence the whole process of atherogenesis [11]. Subsequently, the same researchers discovered three functional variants (Asn263Ser, Pro279Leu and Gly283Asp) in exon 7 in approximately 2% of the affected population, but none in unaffected individuals [12]. Thence, *MEF2A* gene has been considered as the first CAD-causing gene to be identified.

The genomic sequence of *MEF2A* gene is highly polymorphic. It is thus of added interest to detect which or how many *MEF2A* genetic variants might have functional potential to affect the final bioavailability of *MEF2A*, and further the development of CAD. In fact, many case-control studies have attempted to investigate the unequivocal effects of *MEF2A* gene on CAD, especially its exon 11, claimed as the most polymorphic locus harboring various substitution and insertion/deletion (indel) polymorphisms such as a common variant (CAG)_n_ polymorphism. However, the results have been inconsistent [13]–[20].

With the improved genotyping technologies and the completion of the human HapMap project, Genome-Wide Association Studies (GWASs) have been developed as an important approach in genetic research. Thus far, a large number of candidate loci conferring risk of or protection from common complex diseases such as CAD have been proposed [21]–[25]. Nonetheless, neither the *MEF2A* locus on chromosome 15q26 nor its adjacent region has been identified in any of the previous GWASs, thus generating debate over the nature of *MEF2A* genetic contribution to individual susceptibility to CAD.

To elucidate the relationship between *MEF2A* gene and its effect on CAD risk, we focused on its exon 11, the highly polymorphic and controversial region, and established a well-characterized case-control study of 1045 sporadic CAD patients and 1008 controls with normal coronary arteries. In addition, we reviewed all available studies reported in the literature to examine the association of the common (CAG)_n_ polymorphism and the rare 21-bp deletion with CAD, and to assess whether variations in study design and study population ethnicity could lead to potential biases and be the sources of between-study heterogeneity.

## Materials and Methods

### Case-control study

#### Ethics Statement

Approval to undertake this study was granted by the Ethics Review Committee of Ruijin Hospital, Shanghai Jiao Tong University School of Medicine and was conducted according to the Declaration of Helsinki Principles. Written informed consents were obtained from each participant at enrollment.

#### Study population

This was a hospital-based case-control study including a total of 2053 unrelated Han Chinese admitted to Ruijin Hospital, Shanghai Jiao Tong University School of Medicine when they were experiencing various symptoms or for a medical checkup from January 2006 to September 2009. All participants underwent coronary angiography and were divided into CAD group and control group according to their angiographic results.

The CAD group contained 1045 sporadic patients aged 65.49±9.83 years, and the diagnosis of CAD was determined angiographically based on the presence of more than 70% stenosis in at least one of the three major coronary arteries or major branches. Patients with simple spasm of coronary arteries, myocardial bridge or other non-coronary atherosclerotic lesions were excluded. The remaining participants (n = 1008), aged 60.23±10.49 years, had normal coronary arteries (NCA) on angiography, formed the control group.

All patients with the 21-bp deletion were followed up every year in a special CAD clinic. At each visit, clinical manifestations and echocardiography were recorded. Adverse events (e.g. hospitalization, cardiac dysfunction, percutaneous coronary intervention, coronary artery bypass grafting, or death) were reported during the visit or through telephone conversation with the patients or their family members. Two trained physicians independently reviewed all medical notes, including emergency department visit forms and hospital medical records.

#### Screening for variations in *MEF2A* exon 11

Blood samples (5 ml) were drawn and genomic DNA was extracted from peripheral blood leukocytes by standard phenol-chloroform extraction. To assess the distribution patterns of the structural variations of *MEF2A* exon 11 in this cohort study, we sequenced the entire exon 11 using the direct DNA sequencing method in all 2053 subjects. Primers were designed by the Primer3 software (http://frodo.wi.mit.edu/cgi-bin/primer3/primer3) according to reference sequence (NM_005920.2). In detail, the sequence of the forward primer was 5′-gca gag gta ctt gca agc cat ctg-3′ and the reverse was 5′-ggt cgg cca agc aca att gga gaa-3′. The sequencing primer was 5′-caa gca caa ttg gag aat gga-3′. Sequences were analyzed using an ABI Prism BigDye Terminator Cycle Sequencing Kit on an ABI Prism 3700 sequencer, version 3.1 (Applied Biosystems, Foster City, CA, USA), as described in detail in Text S1.

### Systematic review and meta-analysis

#### Data sources and search strategies

We collected information via two international searching engines, *viz.* PubMed and Excerpta Medica Database (EMBASE), and two Chinese searching engines, *viz.* Wanfang database (http://www.wanfangdata.com.cn) and China Biological Medicine (CBM) (http://sinomed.imicams.ac.cn/index.jsp) with the last update on July 31, 2011. We restricted search results to papers published in English or Chinese. We combined the subject terms of ‘coronary artery disease or coronary disease or arteriosclerosis or atherosclerosis or myocardial infarction or angina pectoris’ and ‘myocyte enhancer factor 2A’ with either ‘gene’, ‘variation’, ‘variant’, ‘mutation’, ‘polymorphism’ or ‘allele’, which were all MeSH (Medical Subject Headings in the US National Library of Medicine) terms. The “related articles” in the MEDLINE option as well as reference lists of all retrieved studies were also checked for citations of other relevant publications that were not identified initially. All studies were considered potentially eligible if they aimed to investigate the relationship between *MEF2A* genetic polymorphisms and CAD risk. If there were multiple publications from the same study group, the most complete and recent results were extracted. Search results were limited to studies performed in human subjects without country restrictions and ethnic restrictions.

#### Inclusion/exclusion criteria

We enrolled all prevalent case-control or nested case-control or cross-sectional studies in this meta-analysis regardless of sample size, if 1) they explored the association of *MEF2A* genetic polymorphisms with CAD/MI, 2) genotyping had been performed by using validated methods, and 3) they provided the sufficient information on genotype/allele counts or frequencies for estimating odds ratio (OR) and 95% confidence interval (95% CI). We calculated the effect estimate against healthy subjects/NCA controls.

#### Data extraction

Data were extracted independently and entered into separate databases by two authors (Y. Liu and W. Niu) from each qualified study: first author's last name, publication date, population ethnicity, study design, diagnostic criteria, genotyping methods, baseline characteristics of the study population, such as age, gender, history of hypertension and diabetes mellitus, if available, and the number of persons with different alleles in cases and controls and available subgroups. Discrepancies between the two databases were identified by comparison. A third author (W. Jin) checked for them and a consensus was reached after discussion. For consistency, continuous variables such as age were uniformly expressed as mean ± standard deviation (S.D.)

#### Statistical analysis

For our case-control study, database management and statistical calculation were conducted using SPSS version 13.0 (SPSS Inc., Chicago, Illinois, USA). The Student's *t*-test for continuous variables and the *χ^2^*-test for categorical ones were used to test differences between cases and controls, OR of CAD risk and their 95% CI were calculated as well.

Hardy-Weinberg equilibrium calculations were performed with the Arlequin program (http://anthro.unige.ch/software/arlequin). The Haplo.stats package (version 1.4.0) in the R statistical computing software (http://www.r-project.org) was used to analyze haplotype-based association study. Two-tailed *P*<0.05 was accepted as statistically significant.

In the following meta-analysis, pooled association relating (CAG)_n_ polymorphism to CAD risk was performed by the Review Manager software (version 5.0.19; http://www.cc-ims.net/revman/download). Using the most common type (CAG)_9_ allele as a reference, comparisons of other (CAG)_n_ alleles between cases and controls were expressed in the form of OR and 95% CI. The allele effects were estimated using the model-free approach, where no assumption about genetic models was required. In addition, stratification analyses were conducted to seek more narrowly drawn subsets of the studies such as different genotyping methods, population origins and study designs. We implemented the random-effects model using the method of DerSimonian and Laird, instead of fixed-effects model, to bring the individual effect-size estimates together, and the estimate of heterogeneity was analyzed by the Mantel-Haenszel method [26]–[28].

The presence of between-study heterogeneity across all eligible comparisons was calculated using the *χ^2^*-based Cochrane's Q statistic with statistical significance at the level of 0.10 as this statistic has proven to have poor power if there are few studies [28], [29]. Besides, the *I^2^* statistic was documented for the percentage of the observed between-study variability due to heterogeneity rather than chance with the ranges of 0–100% (*I^2^* = 0–25%, no heterogeneity; *I^2^* = 25–50%, moderate heterogeneity; *I^2^* = 50–75%, large heterogeneity; *I^2^* = 75–100%, extreme heterogeneity) [28].

Finally, publication bias was assessed by the fail-safe number (*N_fs_*) of each meta-analysis [30]. If the *N_fs_* was smaller than the number of observed studies for a polymorphism, it is believed that the meta-result might have a significant publication bias. In this study, the *N_fs_* significance was established at *P*<0.05 (*N_fs0.05_* = (ΣZ/1.64)^2^−*k*; where *k* is the number of articles included in each meta-analysis).

## Results

### Clinical characteristics of our study population

The clinical characteristics of our study population are shown in Table S1. Compared with NCA controls, CAD patients were older (*P*<0.001) and more often of the male gender (*P*<0.001). As expected, the CAD group had a higher prevalence of conventional cardiovascular risk factors, including diabetes and dyslipidemia (*P*<0.05). They had higher serum levels of fasting glucose, total cholesterol, triglycerides, and low density lipoprotein-cholesterol, and lower levels of high density lipoprotein-cholesterol. However, the morbidity of hypertension was similar between the two groups.

### Genetic information on our case-control study

Eight variants were identified by sequencing the entire exon 11 in 2053 unrelated Chinese individuals (Table 1). No significant deviation from the Hardy-Weinberg equilibrium was detected for each polymorphism in both CAD patients and NCA controls.

**Table 1:** Genetic variations in *MEF2A* gene exon 11 discovered by sequencing in our study population.

Categories	Variants	AA code^1^	MAF (allele frequency, %)		*P value; OR [95% CI]*
			CAD Cases(n = 1045)	Controls(n = 1008)	
STR	(CAG)_n_ (1257–1290)	(Q)_n_ (n = 4–15)	More details in Table 4.		
Deletion	CCG (1291–1293)	P deletion (n = 4 or 5, 431/432)	111(5.3)	109 (5.4)	0.892; 1.019 [0.777, 1.337]
	CCA (1297–1299)	P deletion (433/434)	0	1(0.05)	
	21-bp deletion (1303–1337)	QPPQPQP deletion (434–446 AA)	5(0.2)	0	
SNP	A1299G	P433P	1(0.05)	1(0.05)	0.980; 1.036 [0.065, 16.571]
	C1303T	P435S	9 (0.4)	5 (0.2)	0.315; 0.574 [0.192, 1.717]
	G1305A	P435P	92 (4.4)	91 (4.5)	0.867; 0.975 [0.725, 1.312]
	G1353T	G443G	693 (33.2)	689 (34.2)	0.490; 1.047 [0.920, 1.191]

1 Q = Gln; P = Pro; S = Ser; G = Gly.

STR: short tandem repeat polymorphism; SNP: single nucleotide polymorphism; AA: amino acid; MAF: minor allele frequency; OR: odds ratio; 95%CI: 95% confidence interval.

The number of the CAG triplet repeats (polyglutamine tandem repeats, *(Q)_n_*) spanned from 4 to15, and the majority of individuals had 9–11 repeats. Shown in Table 2 are the allele distributions of (CAG)_n_ polymorphism. No statistical significance was observed (*P* = 0.347) for the allelic association of this polymorphism with CAD, and the distribution of genotypes was also similar in two groups (data not shown).

**Table 2:** The baseline characteristics of all the studies relevant to (CAG)_n_ polymorphism in this meta-analysis.

Ref. no	Study	Years	Ethnicity	Status	Enrollment criteria		Genotyping methods	Study size		Cases (allele frequencies, %)				Controls (allele frequencies, %)				*P value*
					Cases	Controls		Cases (n)	Controls (n)	9	10	11	others	9	10	11	others	
17	Weng et al.	2005	White (Canadian)	premature CAD/MI	Angiography confirmed CAD (>50% stenosis), MI or CABG	Symptom investigation	Sequence	287	296	197(32.8)	76(12.7)	292(48.7)	9(1.5)^1^	217(36.2)	80(13.3)	288(48.0)	7(1.2)	0.787
31	Yuan et al.	2006	Asian (southern Chinese)	CAD/MI	Angiography confirmed CAD (>50% stenosis), MI or CABG	Normal angiography	PCR-SSCP	175	228	85(24.3)	66(18.9)	185(52.9)	14(4.0)^2^	106(23.2)	85(18.6)	246(53.9)	19(4.2)	0.985
13	González et al.	2006	White (Spanish)	MI	Angiography confirmed CAD (>30% stenosis), MI	Symptom investigation	Sequence	211	301	136(32.2)	68(16.1)	216(51.2)	2(1.0)^3^	184(30.6)	97(16.1)	316(52.5)	5 (0.8)	>0.05
14	Han et al.	2007	Asian (Northern Chinese)	CAD/MI	Angiography confirmed CAD (>50% stenosis), CABG or MI	Normal angiography	PCR-SSCP	378	348	276(36.5)	158(20.9)	306(40.5)	16(2.1)^4^	158(22.7)	146(21.0)	362(52.0)	30(4.3)	0.001
32	Gulec et al.	2008	Turk	premature MI	MI	Symptom investigation	PCR-SSCP	69	87	70(51.0)	_	62(44.5)	6(4.5)^5^	58(33.5)	_	98 (56.0)	18(10.5)	>0.05
33	Lieb et al.	2008	White (German)	premature MI	MI	Symptom investigation	PCR-SSCP	543	1190	393(36.2)	164(15.1)	510(47.0)	19(1.7)	865(36.3)	350(14.7)	1132(47.6)	33(1.4)	0.800
18	Hsu et al.	2009	Asian (Taiwanese)	CAD/MI	Angiography confirmed CAD (>50% stenosis), MI	Symptom investigation	MALDI-TOF MS	258	258	209(40.5)	108(20.9)	166(32.2)	33(6.4)^6^	180(34.9)	119(23.1)	189(36.6)	28(5.4)	0.179
19	Dai et al.	2010	Asian (Northern Chinese)	CAD	Angiography confirmed CAD (≥75% stenosis)	Normal angiography	Sequence	835	304	675(40.4)	319(19.1)	551(33.0)	125(7.5)^7^	254(41.8)	110(18.1)	213(35.0)	31(5.1)	0.052
	This study		Asian (Southern Chinese)	CAD/MI	Angiography confirmed CAD (≥70% stenosis)	Normal angiography	Sequence	1045	1008	812(38.9)	458(21.9)	670(32.1)	150(7.2)^8^	809(40.1)	434(21.5)	646(32.0)	127(6.3)	0.374

CAD: coronary artery disease; MI: myocardial infarction; PTCA: percutaneous coronary angioplasty; CABG: coronary artery bypass grafting; PCR: polymerase chain reaction; SSCP: single strand conformational polymorphism analysis; MALDI-TOF MS: matrix-assisted laser desorption/ionization time-of-flight mass spectrometry.

1 Number of (CAG)_n_ repeats: 9, 10 or 11 repeats; other, alleles with 5–8, 12, or 13 repeats.

2 Number of (CAG)_n_ repeats: 9, 10 or 11 repeats; other, alleles with 4, 7 or 8 repeats.

3 Number of (CAG)_n_ repeats: 9, 10 or 11 repeats; other, alleles with 6, 7, 8, 12, or 13 repeats..

4 Number of (CAG)_n_ repeats: 9, 10 or 11 repeats; other, alleles with 5, 7, 8, or 12 repeats.

5 Number of (CAG)_n_ repeats: other, alleles with 5, 6, or 7 repeats.

6 Number of (CAG)_n_ repeats: 9, 10 or 11 repeats; other, alleles with 4, 5, 7, 8, 12–15 repeats.

7 Number of (CAG)_n_ repeats: 9, 10 or 11 repeats; other, alleles with 4, 5, 7, 8, 12, 14, or 15 repeats.

8 Number of (CAG)_n_ repeats: 9, 10 or 11 repeats; other, alleles with 4–8, 12–15 repeats.

Comparison between (CAG)_9_ repeats and other types repeats performed by the Review Manager software; OR: odds ratio; 95%CI: 95% confidence interval.

Closely following the (CAG)_n_ polymorphism was the CCG triplet repeats varying between 4 and 5 proline tandem repeats (*P_4_* or *P_5_*). More than 95% of individuals in both CAD patients and NCA controls contained five prolines, and the frequencies of (CCG)_n_ allele and genotypes yielded no significant differences between two groups (data not shown). Additionally, a CCA deletion resulting in lack of one proline amino acid, located adjacent to (CCG)_n_ site, was detected only in one unaffected subject.

Interestingly, the 21-bp deletion was found only in five independent CAD patients, and none in NCA subjects. In this cohort, they all had some traditional CAD risk factors, including dyslipidemia, hypertension and family history of cardiovascular diseases; three showed severe lesion in the left main coronary artery and two were diagnosed with premature CAD. After a 5-year follow up, one died of sudden cardiac death, one took stent treatment and three underwent coronary artery bypass grafting (Table S2).

Besides the aforementioned four variants, we identified three synonymous SNPs (A1299G, G1305A, and G1353T) and one non-synonymous SNP (C1303T) in exon 11. The A1299G and C1303T polymorphisms were rare SNPs with minor allele frequencies being 0.1% and 0.4% among NCA controls, respectively. As a result, none of these four SNPs were associated with CAD (data not shown).

After dropping four rare variations (21-bp deletion, CCA deletion, A1299G and C1303T polymorphisms), we evaluated the remaining four common variants for further haplotype analysis. Using the most common haplotype (Q)_9_-*P_5_*-G-T (in order of (CAG)_n_, (CCG)_n_, G1305A and G1353T) (26.0% in NCA controls) as the baseline for the comparison of the rest, the differences in haplotype distributions between the two groups did not achieve nominal significance (data not shown).

### Meta-analysis results of (CAG)_n_ polymorphism

**Figure 1:**
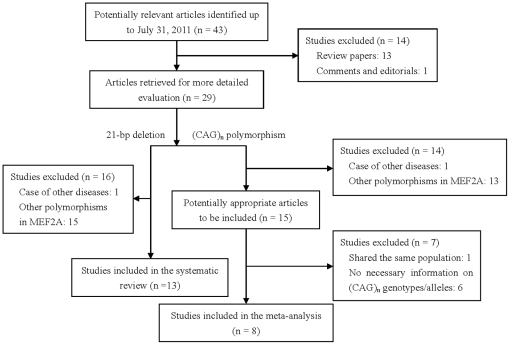
Flow chart of studies identified through the systematic literature search.

The initial search strategy retrieved forty-three relevant articles in English (n = 24) and Chinese (n = 19), in which the effect of *MEF2A* gene variations on CAD was evaluated. A total of fifteen studies met selection criteria, whereas only eight studies [13], [14], [17]–[19], [31]–[33] were tailored to the inclusion criteria in the meta-analysis because six [15], [16], [20], [34]–[36] lacked the necessary information on (CAG)_n_ genotypes/alleles, and one shared the same population [19], [37]. Twenty-five studies, including fourteen review papers, comments and editorials, and fourteen relating to other diseases or polymorphisms in MEF2A gene, were excluded for the final analysis. The flow chart of study selection was summarized in Figure 1. Therefore, data from nine studies, including the present study, totaling 3801 CAD patients and 4020 controls were finally identified in the meta-analysis. Of these, five studies were carried out on Chinese (including this study, 61.85%) [14], [18], [19], [31], three on Whites (36.16%) [13], [17], [33] and one on Turks (1.99%) [32].

**Figure 2:**
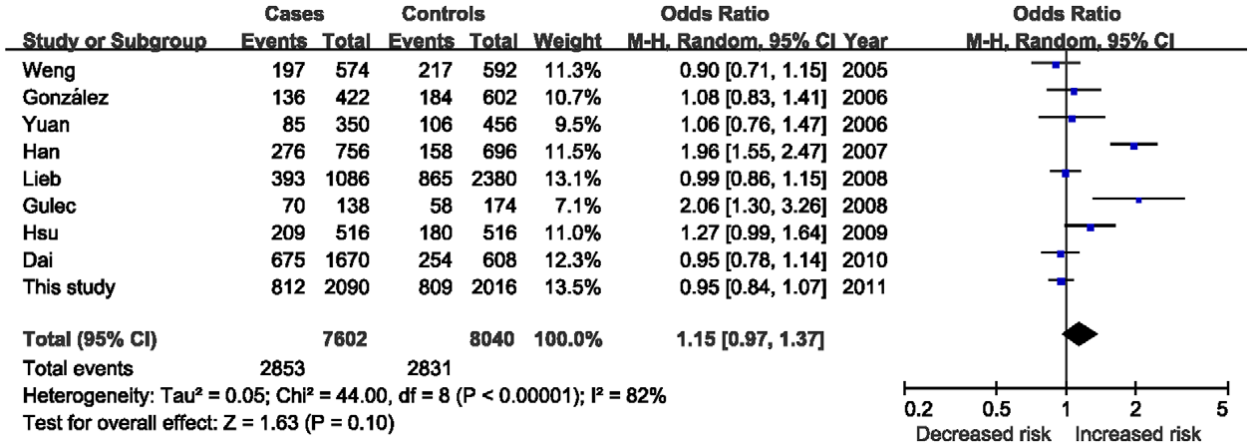
The comparison of the MEF2A (CAG)_9_ allele versus other alleles (with 4–8, 10–15 repeats) under a random effects model.

The demographics and clinical features of all eligible studies are summarized in Table 2. The sample sizes ranged from 156 to 2061. The percentage of males ranged from 72.4% to 87.4% in CAD patients and 49.4% to 79.8% in controls. The mean age was greater than 56 years old in CAD patients and 51 in controls. All studies had allele data of (CAG)_n_ polymorphism except for two with only genotype counts [14], [32]. Seven studies provided information on this polymorphism associated with CAD/MI, and two with MI only [32], [33]. The (CAG)_9_ allele frequency differed widely in diverse ethnic groups. In Whites, the frequencies were in the ranges of 32.2% to 36.2% for CAD cases and 30.6% to 36.3% for controls, which were lower than that in Chinese ranging from 24.3% to 40.5% for cases and 22.7% to 41.8% for controls. In contrast, the Turks had a higher frequency of 51% for cases and 33.5% for controls (Table 2).

As shown in Figure 2, compared with other tandem repeats carriers, those with the (CAG)_9_ allele yielded a non-significant 15% increased risk for CAD (95% CI = 0.97–1.37, *P* = 0.1) under a random-effects model. Whereas, of nine studies, only two individual OR estimates showed a higher risk of CAD that was statistically significant for (CAG)_9_ allele compared with other alleles (Han et al.: OR = 1.96, 95% CI = 1.55–2.47, *P*<0.001; Gulec et al.: OR = 2.06, 95% CI: 1.30–3.26, *P* = 0.002, respectively).

However, statistically significant heterogeneity was evident in most subgroups, according to covariates identified by our qualitative assessment (Table 3). In view of genotyping methods, we classified the nine studies into sequence (including this study) [13], [17], [19]/other (matrix-assisted laser desorption/ionization time-of-flight mass spectrometry, MALDI-TOF MS) [18] and PCR-SSCP (single-strand conformational polymorphism analysis) [14], [31]–[33] groups. There was no significant heterogeneity in the sequence/other group (OR = 1.00, 95% CI: 0.90–1.11; *P_heterogeneity_* = 0.25). In comparison, studies in PCR-SSCP group were heterogeneous (OR = 1.41, 95% CI: 0.94–2.12; *P_heterogeneity_*<0.00001). However, differences in *MEF2A* genotyping methods did not affect the overall results materially.

**Table 3:** Meta-analysis of the effect of (CAG)_9_ allele on CAD risk according to potential sources of heterogeneity.

	Studies (cases/controls), N (n/n)	(CAG)_9_ repeats carriers	
		Overall effect (Z, OR [95%CI], *P value*)	Heterogeneity (*I^2^*, *P value*)
Genotyping methods			
Sequence/other	5 (2636/2167)	0.07, 1.00 [0.90, 1.11], 0.94	26%, 0.25
PCR-SSCP	4 (1165/1853)	1.66, 1.41 [0.94, 2.12], 0.10	90%, <0.00001
Control selection			
Symptom investigation	5 (1368/2132)	1.24, 1.13 [0.93, 1.38], 0.21	68%, 0.01
Normal angiography	4 (2433/1888)	0.90, 1.16 [0.84, 1.61], 0.37	90%, <0.00001
Ethnic			
White	3 (1041/1787)	0.22, 0.99 [0.88, 1.11], 0.82	0%, 0.62
Chinese	5 (2691/2146)	1.22, 1.18 [0.90, 1.54], 0.22	88%, <0.00001
Turk	1 (69/87)	3.08, 2.06 [1.30, 3.26], 0.002	N/A

CAD: coronary artery disease; PCR: polymerase chain reaction; SSCP: single strand conformational polymorphism analysis.

After stratification by control selection criteria based on clinical symptoms or coronary angiographic data, significant heterogeneity was observed in both the NCA group and the symptom investigation group (*P_heterogeneity_* = 0.01 and <0.00001, respectively). Moreover, negative associations persisted across all comparisons.

We divided the population into three groups by ethnicity, Chinese (including this study) [14], [18], [19], [31], White (Spanish, German and Canadian) [13], [17], [33] and Turk [32]. Although, there was no evidence of heterogeneity in White population (OR = 0.99, 95% CI: 0.88–1.11; *P_heterogeneity_* = 0.62), it was significant in Chinese population possibly due to the wide spectrum of (CAG)_9_ allele (OR = 1.18, 95% CI: 0.90–1.54; *P_heterogeneity_*<0.00001). Since there was only one study performed in Turks with a relatively small sample size (n = 156), the risk estimate showed a significant higher risk of (CAG)_9_ allele with CAD (*P* = 0.002), and there was no difference in the pooled risk estimates.

To assess publication bias, we calculated the fail safe number (N*_fs_*) at the level of 0.05 for each comparison. The N*_fs0.05_* values for all the comparisons were greater (62.63) than the number of studies (n = 9) included in this meta-analysis. Therefore, no evidence showed publication bias for association between *MEF2A* gene (CAG)_n_ polymorphism and CAD susceptibility.

### Systematic review of the 21-bp deletion

Of the forty-three potentially relevant studies and the present study, fourteen were eligible for a systematic review of the 21-bp deletion and CAD risk, and thirty studies were excluded (Figure 1). Three of these were family-based studies and the remaining eleven used a hospital-based case-control design (Table 4). Of the latter, four studies (including this study) [14], [19], [38] had used coronary angiography as critical criteria for classification the enrollments, and four studies (including this study) involved more than 1000 subjects in controls [13], [17], [20]. Seven studies were conducted on Whites (53.85%) [11], [13], [17], [20], [33], [34], [38], six on East Asian populations (including this study, 38.46%) [14], [16], [18], [19], [39] and one on Turks (7.69%) [32]. The frequency of the 21-bp deletion in sporadic patients differed substantially, from 0.09% to 1.92%, mainly 0.16% in Whites [20], [38] and 0.65% in Asian (including this study) [16], [19], [39], all were less than 5%. Two studies on Whites [17], [20] and one on Japanese [16] confirmed the 21-bp deletion in controls (not angiographically tested), and the frequency was 0.12% and 0.51%, respectively. The overall frequency of the 21-bp deletion was approximately 0.2% in the combined populations of studies published to date.

**Table 4:** Systematic review of the association between 21-bp deletion and CAD/MI.

Ref. no	Study	Ethnicity	Study design	Genotyping methods	Study size		21-bp deletion	
					Cases	Controls	Cases	Controls
11	Wang et al. (2003)	White (American)	family-based study	Sequence	a single large CAD/MI family		in all ten living CAD/MI members (3 female, 6 with MI, 3 with CABG, 1 with premature MI)	
16	Kajimoto et al. (2005)	Asian (Japanese)	case-control study	Sequence	379 MI	589	3, no detailed data	3, not angiographically tested
17	Weng et al. (2005)	White (Canadian)	case-control study	Sequence	300 premature CAD	1821	none	3 elderly subjects in a 71-year-old female kindred; a 45-year-old obese male with diabetes; a 45-year-old normal-weight male; all of them not angiographically tested
13	González et al. (2006)	White (Spanish)	case-control study	Sequence	483 MI	1189	none in any individuals	
34	Horan et al. (2006)	White (Irish)	family-based study	Sequence	1481 individuals from 573 families with premature CAD		none in any individuals	
39	Li et al. (2006)	Asian (Northern Chinese )	case-control study	PCR-SSCP	156 CAD/MI	93	a 40-year-old male; a 69-year-old male with dyslipidemia, family history of CAD and diabetes; a 50-year-old male with dyslipidemia and smoking; all of them had three-vessel disease	none
14	Han et al. (2007)	Asian (Northern Chinese)	case-control study	PCR-SSCP	378 CAD	348	none in any individuals	
32	Gulec et al. (2008)	Turk	case-control study	PCR-SSCP	69 premature MI	87	none in any individuals	
33	Lieb et al. (2008)	White (German)	family-based study	PCR-SSCP	23 representative individuals with familial MI		none	
18	Hsu et al. (2009)	Asian (Taiwanese)	case-control study	MALDI-TOF MS	258 CAD/MI	258	none	
20	Guella et al. (2009)	White (Italian)	case-control study	Sequence	3127 CAD/MI	3083	5 obese males with premature MI (4 with smoking, 2 with dyslipidemia)	a 55-year-old woman, not angiographically tested
19	Dai et al. (2010)	Asian (Northern Chinese)	case-control study	PCR-SSCP	257 CAD	154	1, no detailed data	none
38	Maiolino et al. (2011)	White (Italian)	case-control study	FRET and HRMA	1079 CAD	301	a 52-year-old male with early onset three vessels CAD, hypertension, smoking, family history of MI and sudden death	none
	This study	Asian (Southern Chinese)	case-control study	Sequence	1045 CAD	1008	5, more details in table 3	none

CAD: coronary artery disease; MI: myocardial infarction; PCR: polymerase chain reaction; SSCP: single strand conformational polymorphism analysis; MALDI-TOF MS: matrix-assisted laser desorption/ionization time-of-flight mass spectrometry; FRET: fluorescence resonance energy transfer technology; HRMA: high resolution melting analysis.

As shown in Table 4, more than half of patients bearing the deletion either suffered severe CAD who had undergone percutaneous coronary intervention or coronary artery bypass grafting, or had some traditional CAD risk factors, such as hypertension, diabetes, dyslipidemia, smoking, drinking, and/or family history of CAD/MI or sudden death. But, the results of different studies were inconsistent. Hsu et al. [18], [33] failed to detect this rare variant in CAD patients. In contrast, Weng et al. [17] discovered this deletion in unaffected individuals rather than in CAD patients. However, Kajimoto et al. [16], [20] reported this variant in both CAD patients and controls, and González et al. [13], [14], [32], [34] did not reveal this deletion in any subject of the study population.

## Discussion

In the present study, we verified eight variants in *MEF2A* exon 11 and found that the most conspicuously heterogeneous variant was the (CAG)_n_ polymorphism, while the other seven were all downstream of this polymorphism within 100 bp. Such intense variation in the context of a single exon led us to explore the link of *MEF2A* genetic polymorphisms to CAD/MI. A possible explanation might be the remarkable diversity embedded in (CAG)_n_ polymorphism. We therefore carried out a rigorously-designed case-control association study focusing on *MEF2A* exon 11 in southern Chinese and reviewed all available information regarding the relationship between this genetic hotspot and sporadic CAD/MI from the literature. To the best of our knowledge, this is the first meta-analysis seeking to clarify the association of *MEF2A* gene (CAG)_n_ polymorphism with CAD risk.

Data from our case-control study, which was in concordance with most previous observations, and in combination with all other eight studies involving a total of 3801 CAD patients and 4020 controls, failed to show a significant association between (CAG)_n_ polymorphism and CAD susceptibility, even upon stratification by race (mainly Whites and Chinese). This meta-analysis had sufficient statistical power [40] to detect such genetic effect. Therefore, it is reasonable to surmise that either (CAG)_n_ polymorphism itself exhibits null association with CAD, or its effect on CAD is small and depends on neighboring variants that compensate or dilute the variation under study.

Noteworthy, one study conducted by Han et al. [14] in a small northern Chinese cohort showed a positive and independent association of (CAG)_9_ allele with an increased risk and severity of CAD, while data from Dai et al. [19] in another 1139 northern Chinese cohort displayed a marginal significance (P = 0.052). However, in our present study, we failed to replicate this association in southern Chinese, which was in line with two other Chinese populations [18], [31] and in agreement with the pooled estimate of this meta-analysis. Except for the differences in diet and climate between northern and southern China, this discrepancy might be caused by misgenotyping as discussed by Hsu et al. [18]. All participants in Han's study were homozygous for (CAG)_n_ polymorphism; the phenomenon was not compatible with the situation expected from random mating. After separating analyses by genotyping methods, we found that heterogeneity between studies in the PCR-SSCP group was higher than the overall estimate; however, there was no indication attributable to the diversity between different experimental methods. Nevertheless, applying appropriate genotype techniques remain an open question.

Moreover, it is well known that CAD is frequently asymptomatic and the diagnosis relies on coronary angiography. However, the definition of controls was debatable in a number of the available studies. Some controls were enrolled according to their clinical symptoms and should have been properly defined as “uncertain phenotype” [41], whereas some were on the basis of explicit coronary angiographic results. Thus we cannot exclude the possibility that the apparently healthy elderly controls had underlying CAD, and so confuse and bias the study conclusions. Therefore, our meta-analysis pinpointed the different selective criteria of controls as a potentially significant source of between-study heterogeneity. Nevertheless, deviation in the controls did not appear to be a significant source of between-study heterogeneity. Although this observation seems counterintuitive in terms of selective criteria, considering the relative small sample sizes even in the present meta-analysis and the possibility that (CAG)_n_ polymorphism might not be a major contributing locus or have limited values to assess an exact role of *MEF2A* in CAD/MI, we maintain that application of coronary angiographic criteria for controls is preferable, and the proper phenotype discrimination is critical in any genetic association study.

Meanwhile, we observed the wide divergence of (CAG)_n_ repeats across different populations. Specifically, the high versus low frequency of (CAG)_9_ allele was nearly double in both CAD patients and controls, suggesting a possible role of differences in genetic background and the environment in which the populations live. Of note, there was only one eligible Turkish population [32], and there was statistical evidence of heterogeneity between subgroups only in this population. It was likely that this positive association between the (CAG)_9_ allele and CAD in the Turkish study might be due to chance or confounding, for its sample size was rather small (n = 156) and its deviation would have little or no effect on our null results. We agree that more studies in diverse ethnic/racial groups are required to draw a firm conclusion.

It was noteworthy that we identified the 21-bp deletion only in affected individuals, which was consistent with the results from Wang et al. [11], [19], [38], [39], but which are contradicted by observations in other [13], [14], [16]–[18], [20], [32]–[34]. Considering that the 21-bp deletion was firstly identified in an exceptional CAD family displaying an autosomal-dominant pattern of inheritance and no families in the specific context were ever available for genetic linkage analysis thus far, the molecular case-control association studies of unrelated samples have become the alternative research strategy, but the results were inconsistent. Researchers have argued strongly against this deletion as a causal variant in the mechanisms of CAD pathopoiesis [41], [42]. In view of possibly different genetic profiles and clinical features, we cannot jump to a conclusion regarding the cosegregation of the 21-bp deletion with CAD until validation in well-designed, large cohort studies. On the other hand, the susceptibility of patients with the 21-bp deletion to CAD supports the common-disease rare-variant (minor allele frequency less than 5%) hypothesis (CDRV) rather than the common-disease common-variant hypothesis (CDCV) [43], [44]. There exists a ‘common-variant, small-effect’ model and also the possibility of a ‘rare-variant, large-effect’ model [34], the question is not which model is correct, but rather what is the relative contribution of each [43], [45]–[48]. Although our findings add potent evidence favoring the association of the 21-bp deletion with CAD, the possibility of a founder effect from a common variant, such as (CAG)_n_ polymorphism, cannot be ruled out. The challenge here is to decide which observed variants in *MEF2A* gene could be considered as a susceptibility or causal mutation in CAD. Our data indicate that the rare 21-bp deletion might have a more compelling effect on CAD than the common (CAG)_n_ variant, and *MEF2A* genetic variants might, therefore, be a rare but specific cause of CAD/MI. Needless to say, a composite effect encompassing all influential *MEF2A* genetic variants remains to be determined.

Finally, some limitations of this study should be acknowledged when interpreting the results. Firstly, it is recognized that differences in study design, genetic heterogeneity and statistical methods made it harder to estimate the exact underlying genetic contribution to disease susceptibility. Moreover, some large-scale studies [15], [20], [34] could not be included in our meta-analysis because of their incomplete raw data. These could have potentially introduced additional factors and influenced our results. Secondly, most of the enrolled study samples, including our affected population, were all survivors of CAD, as we could not evaluate those who did not survive. Thirdly, considering the complex interplay between the *MEF2A* gene and others that operate in the same pathway, the single-locus based case-control study and meta-analysis preclude the possibility of gene-gene and gene-environment interactions and may not reveal the full picture. Although there was no evidence showing publication bias in our overall meta-analysis, considering the above limitations, further studies with larger sample size and different ethnic compositions, which typically considered as small or moderate effects, are warranted to avoid study bias.

In conclusion, our case-control study and the following meta-analysis provide no convincing evidence for the genetic involvement of *MEF2A* gene (CAG)_n_ polymorphism in CAD. However, we suggested that the 21-bp deletion might be a rare but specific cause of CAD. As few studies are available in this field and current evidence remains limited, this conclusion requires further confirmation by well-designed prospective studies with adequate methodological quality and properly controlling for possible confounds, particularly different genetic approaches, homogeneous CAD patients and well-matched controls, gene-gene and gene-environment interactions, and multiethnic groups.

## Supporting Information

Text S1 Sequence analysis.
(DOC)

Table S1 The clinical characteristics of our study population.
(DOC)

Table S2 Demographics of the CAD patients with 21-bp deletion in our study population.
(DOC)
