# Supplementary material for: Variants in Exon 11 of MEF2A Gene and Coronary Artery Disease: Evidence from a Case-Control Study, Systematic Review, and Meta-Analysis
Source: PLoS One. 2012 Feb 21;7(2):e31406. doi: 10.1371/journal.pone.0031406 (PMC3283621; doi:10.1371/journal.pone.0031406)
Supplement: Text S1 — Sequence analysis. (DOC) [file pone.0031406.s001.doc]

Text S1 Sequence analysis

PCRs were carried out in a total volume of 15 μL containing 0.3 mM of each deoxynucleoside triphosphate (dNTPs), 10 mM Tris-HCl (pH 8.3), 100 mM KCl, 2 mM MgCl_2_, 0.2 μM of each primer, 20 ng genomic DNA, and 1.2 U Taq DNA polymerase (TaKaRa, Otsu, Shiga, Japan). Cycling conditions were as follows: 94°C for 3 min, followed by 10 cycles of 94°C for 30 s, 67°C for 30 s with a 0.5°C decrement of the annealing temperature per cycle and 72°C for 45 s, followed by 30 cycles of 94°C for 30 s, 62°C for 30 s and 72°C for 30 s, followed by 72°C for 8 min. Preparation of DNA for sequencing included incubation of PCR products with 2 U of CIAP (TaKaRa, Dalian, China) and 4 U of exonuclease I (Fermentas, Vilnius, Lithuania) at 37 °C for 30 min, followed by heat inactivation at 95°C for 10 min.
