# Supplementary material for: Variants in Exon 11 of MEF2A Gene and Coronary Artery Disease: Evidence from a Case-Control Study, Systematic Review, and Meta-Analysis
Source: PLoS One. 2012 Feb 21;7(2):e31406. doi: 10.1371/journal.pone.0031406 (PMC3283621; doi:10.1371/journal.pone.0031406)
Supplement: Table S1 — The clinical characteristics of our study population. (DOC) [file pone.0031406.s002.doc]

Table S1 The clinical characteristics of our study population.

|  | Controls (n=1008) | CAD Cases (n=1045) | *P value* |
| --- | --- | --- | --- |
| Age (years)^1^ | 60.23±10.49 | 65.49±9.83 | < 0.001 |
| Males (%) | 533(52.9) | 768(72.9) | < 0.001 |
| Systolic blood pressure (mmHg) | 131.76±18.1 | 138.61±21.14 | < 0.001 |
| Diastolic blood pressure (mmHg) | 80.66±10.76 | 82.28±12.92 | 0.004 |
| Total cholesterol (mmol/L) | 4.62±0.93 | 4.79±1.13 | 0.001 |
| Triglyceride (mmol/L) | 1.74±0.96 | 1.95±1.28 | < 0.001 |
| High density lipoprotein-cholesterol (mmol/L) | 1.25±0.41 | 1.20±0.48 | 0.016 |
| Low density lipoprotein-cholesterol (mmol/L) | 2.76±0.78 | 2.87±1.00 | 0.015 |
| Fasting glucose (mmol/L) | 5.34±1.16 | 5.79±2.14 | < 0.001 |
| Hypertension (%) | 641(63.6) | 701(66.6) | 0.156 |
| Diabetes mellitus (%) | 120(11.9) | 254(24.1) | < 0.001 |

^1^Age at onset for cases and at examination for controls.

CAD: coronary artery disease
