# Supplementary material for: Variants in Exon 11 of MEF2A Gene and Coronary Artery Disease: Evidence from a Case-Control Study, Systematic Review, and Meta-Analysis
Source: PLoS One. 2012 Feb 21;7(2):e31406. doi: 10.1371/journal.pone.0031406 (PMC3283621; doi:10.1371/journal.pone.0031406)
Supplement: Table S2 — Demographics of the CAD patients with 21-bp deletion in our study population. (DOC) [file pone.0031406.s003.doc]

Table S2 Demographics of the CAD patients with 21-bp deletion in our study population.

| Individual ID | Sex | On-set age | Clinical diagnosis for CAD | Coronary angiography diagnosis | CAD traditional risk factors | 5-year follow up |
| --- | --- | --- | --- | --- | --- | --- |
| NO.1 | M | 50 | unstable angina | LAD (80%) | Hypertension | stent, alive |
| NO.2 | M | 58 | myocardial infarction | LM (90%), LAD (100%) | Hypertension, Smoking, Drinking, Family history of hypertension (father) | CABG, alive |
| NO.3 | F | 79 | unstable angina | LAD (100%), RCA (75%) | Hypertension, Dyslipidemia | stent, cardiac sudden death in 2009 |
| NO.4 | F | 50 | unstable angina | LM (100%) | Hypertension, Family history of hypertension (elder sister) | CABG, alive |
| NO.5 | M | 67 | unstable angina | LM (90%), LAD (100%),  LCX (100%), RCA (90%) | Hypertension | CABG, alive |

CAD: coronary artery disease; LM: left main coronary artery; LAD: left anterior descending artery; LCX: left circumflex artery; RCA: right coronary artery; CABG: coronary artery bypass grafting.
